# Hepatitis C-related hepatocellular carcinoma in the era of new generation antivirals

**DOI:** 10.1186/s12916-017-0815-7

**Published:** 2017-03-14

**Authors:** Thomas F. Baumert, Frank Jühling, Atsushi Ono, Yujin Hoshida

**Affiliations:** 1 Inserm, U1110, Institut de Recherche sur les Maladies Virales et Hépatiques, Strasbourg, France; 2 Université de Strasbourg, Strasbourg, France; 3 Institut Hospitalo-Universitaire, Pôle Hépatodigestif, Nouvel Hôpital Civil, Strasbourg, France; 4 Division of Liver Diseases, Department of Medicine, Liver Cancer Program, Tisch Cancer Institute, Graduate School of Biomedical Sciences, Icahn School of Medicine at Mount Sinai, 1470 Madison Ave, Box 1123, New York, NY 10029 USA; 5 Department of Gastroenterology and Metabolism, Applied Life Sciences, Institute of Biomedical and Health Sciences, Hiroshima University, Hiroshima, Japan

**Keywords:** Hepatitis C virus, Hepatocellular carcinoma, Interferon, Direct-acting antivirals, Sustained virologic response

## Abstract

Hepatitis C virus infection is a major cause of hepatocellular carcinoma worldwide. Interferon has been the major antiviral treatment, yielding viral clearance in approximately half of patients. New direct-acting antivirals substantially improved the cure rate to above 90%. However, access to therapies remains limited due to the high costs and under-diagnosis of infection in specific subpopulations, e.g., baby boomers, inmates, and injection drug users, and therefore, hepatocellular carcinoma incidence is predicted to increase in the next decades even in high-resource countries. Moreover, cancer risk persists even after 10 years of viral cure, and thus a clinical strategy for its monitoring is urgently needed. Several risk-predictive host factors, e.g., advanced liver fibrosis, older age, accompanying metabolic diseases such as diabetes, persisting hepatic inflammation, and elevated alpha-fetoprotein, as well as viral factors, e.g., core protein variants and genotype 3, have been reported. Indeed, a molecular signature in the liver has been associated with cancer risk even after viral cure. Direct-acting antivirals may affect cancer development and recurrence, which needs to be determined in further investigation.

## Background

Chronic infection of hepatitis C virus (HCV), estimated to affect more than 150 million individuals globally, has proven a major health problem by causing liver cirrhosis and cancer. Hepatocellular carcinoma (HCC), the major liver cancer histological type, is the second leading cause of cancer mortality worldwide [1]. Interferon-based regimens have been the mainstay of anti-HCV therapy, yielding HCV cure, or sustained virologic response (SVR), in approximately 50% of patients [2]. Recently developed direct-acting antivirals (DAAs), which directly target the viral protease, polymerase, or non-structural proteins, have enabled interferon-free anti-HCV therapies with a revolutionary improvement of SVR rate, approaching or surpassing 90% [3]. Despite the unprecedentedly high antiviral efficacy, access to therapy remains limited, at less than 10% of the total number of HCV-infected individuals, especially in developing countries, due to the high drug costs [4, 5]. In addition, the high frequency of unrecognized HCV infection in the general and specific (e.g., inmates and homeless) populations, estimated at 50% or above of infected individuals, hampers control of the virus even with the commercialization of DAAs [5, 6]. With the 3–4 million newly infected cases each year, it is estimated that the HCV-associated disease burden will remain high in the next decade, even in developed countries [7–9].

Wider use of DAAs has revealed several limitations, including more refractory genotype 1a or 3 virus, emergence of resistant HCV stains with characteristic resistance-associated substitutions, and poorer response in prior non-responders to interferon-based therapies [10]. These findings warrant further development of alternative strategies such as the application of specific DAA combination therapies to target resistant HCV strains, host targeting agents**/**viral entry inhibition, and the development of diagnostic tools to monitor therapeutic progress and success [11–14]. Even after SVR, re-infection can occur in up to 10–15% of patients, especially in high-risk populations such as injection drug users [15–17]. Post-transplantation graft re-infection is also a critical issue given that most liver explants (67%) remain HCV RNA positive in the liver despite undetectable blood HCV RNA [18]. In these clinical scenarios, a prophylactic vaccine is likely to be an effective option. Although HCV vaccine development is challenging due to the high viral genetic variability, promising progress has been made to date [16]. Thus, complementary antiviral approaches including improved and more accessible therapies as well as the development of a prophylactic vaccine will be necessary to achieve impactful global control of infection that leads to HCV eradication, namely a reduction of regional incidence close to zero.

### Effect of HCV cure on HCC development

**Fig. 1:**
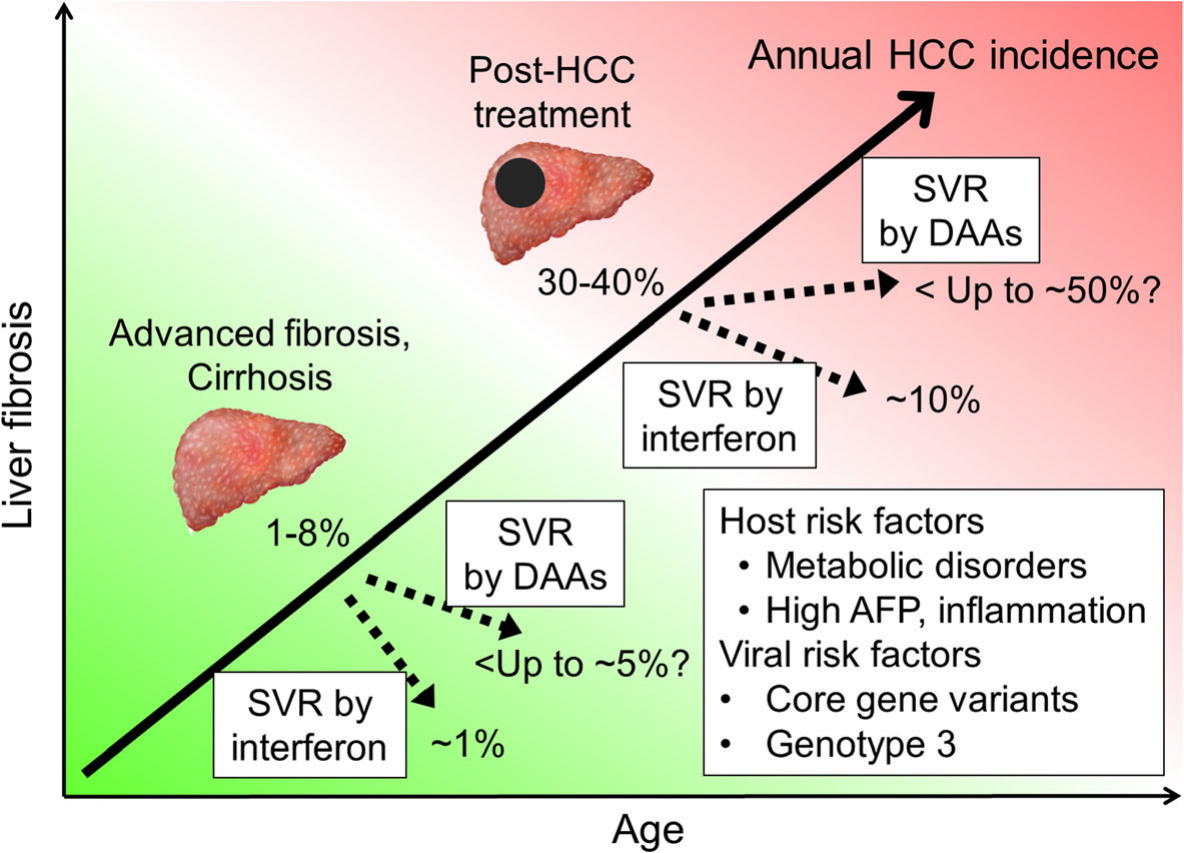
Natural history of HCV-related HCC development and modulation by anti-HCV therapies. Progressive liver fibrosis along with aging gradually increases the risk of hepatocarcinogenesis, which could be further accelerated by several host and viral risk factors. Annual incidences of HCC development and recurrence after DAA-based SVR were estimated from retrospective and prospective studies summarized in Table 1. SVR induced by interferon- or DAA-based anti-HCV therapies may result in distinct post-SVR HCC risk. *AFP* alpha-fetoprotein, *DAA* direct-acting antiviral, *HCV* hepatitis C virus, *HCC* hepatocellular carcinoma, *SVR* sustained virologic response

HCV-induced progressive liver fibrosis and aging are well-established high-risk conditions for HCC development (Fig. 1) [1]. Of note, the highly carcinogenic “field effect” in fibrotic/cirrhotic livers leads to repeated recurrence of de novo HCC tumors even after curative treatment of the initial primary tumors. The effect of achieving an SVR on HCC risk has been reported in multiple retrospective cohorts of patients who were mostly treated with interferon-based therapies (Table 1); these studies consistently showed significant reduction of HCC incidence in SVR patients. A pooled analysis of 12 observational studies with a total of 25,497 patients showed that interferon-induced SVR resulted in an approximately four-fold reduction of HCC risk irrespective of liver disease stage (hazard ratio, 0.24; *P* < 0.001) [19]. Annual HCC incidence in patients with advanced liver fibrosis or cirrhosis and active HCV infection is reported to range from 1% to 8% [1], reducing to 0.07% to 1.2% after achieving an SVR by interferon-based therapies (Table 1). SVR is also implicated in reduction of all-cause mortality, which may alter patient prognosis to the level of the general population, although this is yet to be established [20, 21]. In cirrhotic patients who failed to achieve SVR, subsequent maintenance low-dose interferon treatment reduced annual HCC incidence to 1.2% when compared to 4.0% in untreated patients (hazard ratio, 0.45; *P* = 0.01) [22]. This result suggests that the anti-inflammatory and/or immunomodulatory effects of interferon may have HCC-preventive effects irrespective of HCV presence, although the adverse effects (flu-like symptoms, neuropsychiatric and myelosuppressive effects) hamper its wider use [1]. DAAs are shown to be less toxic, and are expected to overcome the limitations of interferon-based therapies. DAAs were indeed well-tolerated even in compensated and decompensated cirrhosis patients in recent clinical trials [23, 24]. However, their HCC preventive effect is still only partially understood.

**Table 1:** Incidence of post-sustained virologic response (SVR) hepatocellular carcinoma (HCC) development and recurrence

Author (year), Reference	Major race	Country	Type of anti-HCV therapy	n	Follow-up, median, years	Male sex, n (%)	Age, median, years	Advanced fibrosis/cirrhosis, n (%)	Treatment for previous HCC	Reported HCC incidence in SVR, % (interval in years)	Annual HCC incidence in SVR, %	Reported HCC incidence in non-SVR, % (interval in years)	Annual HCC incidence in non-SVR, %	Study design
HCC development														
Akuta (2011) [34]	Asian	Japan	IFN-based	1273	1.1	783 (61.5)	53	109 (8.6)	–	3.2 (5)	0.65	–	–	Retrospective
Chang (2012) [51]	Asian	Taiwan	IFN-based	1271 (including 400 non-SVR)	3.4	661 (75.9)	55.4^a^	355 (27.9)	–	1.2 (3)	0.4	–	–	Retrospective
Huang (2014) [52]	Asian	Taiwan	IFN-based	642	4.4	302 (54.3)	51.4^a^	86 (13.4)	–	5.8 (5)	1.2	–	–	Retrospective
Yamashita (2014) [53]	Asian	Japan	IFN-based	562	4.8	311 (55.3)	57	129 (23.0)	–	3.1 (5)	0.63	–	–	Retrospective
Oze (2014) [54]	Asian	Japan	IFN-based	1425	3.3	727 (51.0)	54.5	118 (11.6)	–	2.6 (5)	0.53	11.7 (5)	2.49	Prospective
Toyoda (2015) [55]	Asian	Japan	IFN-based	522	7.2	292 (55.9)	50.6	27 (5.5)	–	1.2 (5)	0.24	–	–	Retrospective
Wang (2016) [56]	Asian	Taiwan	IFN-based	376	7.6	185 (49.2)	54.1	127 (33.8)	–	1.4 (5)	0.28	–	–	Retrospective
Kobayashi (2016) [57]	Asian	Japan	IFN-based	528	7.3	308 (58.4)	54	78 (14.8)	–	2.2 (5)	0.44	–	–	Retrospective
El-Serag (2016) [36]	Caucasian	USA	IFN-based	10,738	2.8	10,232 (95.3)	53.1^a^	1548 (14.4)	–	0.33 (5)	0.07	–	–	Retrospective
Nagaoki (2016) [58]	Asian	Japan	IFN-based	1094	4.2	585 (53.5)	60	208 (1.9)	–	4.0 (5)	0.82	–	–	Retrospective
Tada (2016) [59]	Asian	Japan	IFN-based	587	14.0	324 (55.2)	50	–	–	4.4 (10)	0.45	14.7 (10)	1.59	Retrospective
Tada (2016) [60]	Asian	Japan	IFN-based	170	14.2	106 (62.4)	52.5	–	–	7.1 (10)	0.74	–	–	Retrospective
van der Meer (2016) [61]	Caucasian	Europe and Canada	IFN-based	1000	5.7	676 (68.0)	52.7	842 (85.0)	–	7.6 (8)	0.99	–	–	Retrospective
Kobayashi (2016) [57]	Asian	Japan	DAAs	77	4.0	34 (44.2)	63	23 (29.9)	–	3.0 (5)	0.62	–	–	Retrospective
HCC development or recurrence														
Conti (2016) [39]	Caucasian	Italy	DAAs	344 (59 had previous HCC, including 30 non-SVR)	0.5	207 (60.1)	63	39 (11.3)	Resection, ablation, TACE	3.2 (0.5)^d^	6.3^d^	–	–	Retrospective
Cheung (2016) [24]	Caucasian	UK	DAAs	317 (18 had previous HCC)	1.3	NA	54	254 (80.1)	Resection, ablation, TACE	5.4 (1.3)	4.27	11.2 (1.3)	9.14	Prospective
HCC recurrence														
Saito (2014) [62]	Asian	Japan	IFN-based	14	3.9	13 (92.9%)	72	12 (85.7)	Resection, ablation	18.0 (3)	6.62	75.3 (3)	46.61	Retrospective
Huang (2015) [63]	Asian	Taiwan	IFN-based	56	4.4	36 (64.3)	61.6	21 (37.5)	Resection, ablation	43.2^b^	–	84.8^b^	–	Retrospective
Kunimoto (2016) [64]	Asian	Japan	IFN-based	40	5.1	35 (87.5%)	65	14 (35.0)	Resection, ablation	23.0 (3)	8.71	56.0 (3)	27.37	Retrospective
Petta (2016) [65]	Caucasian	Italy	IFN-based	57	2.8	41 (72.0)	62	0	Resection, ablation	15.2 (2)	8.24	–	–	Retrospective
Minami (2016) [66]	Asian	Japan	IFN-based	38	–	27 (71.0)	66	0	Ablation	52.9 (2)	37.6	–	–	Retrospective
Conti (2016) [39]	Caucasian	Italy	DAAs	59 (including 6 non-SVR)	0.5	40 (67.8)	72	10 (16.9)	Resection, ablation, TACE	28.8^d^ (0.5)	49.3^d^	–	–	Retrospective
Reig (2016) [40]	Caucasian	Spain	DAAs	58	0.5	40 (69.0)	66.3	5 (8.6)	Resection, ablation, TACE	27.6^d^ (0.5)	47.6^d^	–	–	Retrospective
ANRS study group (2016) [42]	Caucasian	France	DAAs	189 (including 41 non-SVR)	2.2	147 (78.0)	62^a^	152 (80.0)	Resection, ablation, LT	0.73^b,d^	8.76^c,d^	0.66^b,e^	7.92^c,e^	Prospective
ANRS study group (2016) [42]	Caucasian	France	DAAs	13	1.8	11 (85.0)	61^a^	13 (100)	Resection, ablation	1.1^b^	13.3^c^	1.7^b,e^	20.76^c,e^	Prospective
ANRS study group (2016) [42]	Caucasian	France	DAAs	314	–	257 (82.0)	61^a^	49 (15.6)	LT	2.2 (0.5)	4.4	–	–	Prospective
Petta (2016) [65]	Caucasian	Italy	DAAs	58	1.5	40 (69.0)	66.3	2 (4.0)	Resection, ablation	26.3 (2)	15.3	–	–	Retrospective
Minami (2016) [66]	Asian	Japan	DAAs	27	–	18 (67.0)	71	0	Ablation	29.8 (2)	17.7	–	–	Retrospective

When not reported, annual HCC incidence was estimated by using the declining exponential approximation of life expectancy [67]

^a^Mean

^b^100 person-month

^c^100 person-year

^d^Incidence in patients including non-SVR patients

^e^Incidence in patients not treated by anti-HCV therapy

*DAA* direct-acting antiviral, *HCC* hepatocellular carcinoma, *HCV* hepatitis C virus, *IFN* interferon, *LT* liver transplantation, *SVR* sustained virologic response, *TACE* transarterial chemoembolization

### Projected trend of HCV HCC incidence with new generation anti-HCV therapies

HCC is the most rapidly increasing cause of cancer death, with HCV as the major etiology affecting generally more than half of HCC patients in developed countries such as the USA [25]. HCV incidence increases are more prominent in specific subpopulations such as the 1945–1965 birth cohort (baby boomers) in the USA, in whom a 64% incidence was observed between 2003 and 2011; such an incidence is estimated to result in more than one million HCV-related cirrhosis and/or HCC by 2020, with increasing HCC incidence until 2030 [26–28]. In US veterans, HCC incidence has increased by 2.5-fold and mortality has tripled since 2001, driven overwhelmingly by HCV [29]. In a regional population in Australia, in contrast to the decreased incidence of hepatitis B virus (HBV)-related HCC due to clinical implementation of the antivirals, anti-HCV therapies had no impact on HCV-related HCC risk between 2000 and 2014 [30]. Despite the anticipated improvement in SVR rate with wider use of DAAs, model-based simulation studies have predicted further increases of HCC incidence over the next decade – even with SVR rates of 80–90% by DAAs, predicted HCC incidence will continue to increase until 2035 unless the current annual treatment uptake rate (1–3%) is increased by more than five-fold by 2018 [9, 31, 32]. These studies clearly highlight the urgent need for identification of undiagnosed HCV infection by implementing HCV screening programs targeting high-risk populations as well as improved access to new generation anti-HCV therapies with reduced costs and streamlined treatment intake and follow-up [33].

### Post-SVR HCC risk factors

It is noteworthy that SVR does not necessarily mean elimination of HCC risk despite the substantially decreased incidence. In fact, HCC can occur even more than 10 years after successful HCV clearance (Table 1). The annual post-SVR HCC incidence of approximately 1% is still higher than the cancerous conditions in other organs, and the volume of HCC-developing patients will remain substantial given the vast size of the HCV-infected population [1].

Retrospective interrogation of previously treated patients mostly by interferon-based regimens revealed several post-SVR HCC-associated clinical variables, most of which are known HCC risk factors in patients with active HCV infection (Table 2). More advanced liver fibrosis as well as biochemical or imaging surrogates of histological fibrosis (e.g., serum albumin, platelet count, fibrosis-4 index, aspartate aminotransferase-to-platelet ratio index, elastography-based liver stiffness) before and/or after antiviral treatment are the most prominent features associated with higher post-SVR HCC risk. Older age, alcohol abuse, accompanying metabolic disorders (especially diabetes), and persisting hepatic inflammation, e.g., high aspartate aminotransferase, were also associated with HCC risk. Serum alpha-fetoprotein levels pre- and post-SVR have also been implicated as a risk indicator, with relatively low cut-off values ranging from 5 to 20 ng/mL. In addition to the host factors, post-SVR HCC-associated pre-treatment viral factors have been identified, suggesting that HCV leads to irreversible changes in cellular signaling via mechanisms such as epigenetic activation or imprinting, which continue to drive carcinogenesis even after viral clearance. A variant in genotype 1b HCV core protein, Gln70(His70), was associated with increased HCC incidence post-SVR, with a hazard ratio of 10.5, in a cohort of 1273 interferon-treated Japanese patients [34]. Interestingly, the variant can induce cancer-related transcriptional dysregulation in an HCV-infectious cell system [35]. HCC risk association of genotype 3 was also found in a cohort of 10,817 US veterans [36]. A further study suggested differences in molecular aberrations in HCC tumors from SVR livers compared to tumors in livers with active HCV infection, which may represent SVR-specific mechanisms of carcinogenesis [37].

**Table 2:** Host and viral risk factors for post-sustained virologic response (SVR) hepatocellular carcinoma (HCC) development (summarized from multivariable Cox regression models)

Risk factor		Variable	*n*	Country	Follow-up, median, years	Hazard ratio	95% CI	*P* value	Reference
Host factor	Fibrosis								
	Pre-SVR								
		Histological stage F2-4	562	Japan	4.8	10.7	2.2–192.1	<0.001	[53]
		Histological stage, F3-4	1273	Japan	1.1	9.0	2.3–35.2	0.002	[34]
		Histological stage, F3-4	1094	Japan	4.2	3.2	1.6–7.2	<0.001	[58]
		Histological stage, F3-4	376	Taiwan	7.6	12.8	1.6–101.9	0.021	[56]
		Histological stage, F3-4	871	Taiwan	3.4	4.0	1.5–10.7	0.007	[51]
		Platelet, < 150 × 10^3^/mm^3^	1056	Japan	4.7	2.8	1.1–7.2	0.04	[68]
		Platelet, < 150 × 10^3^/mm^3^	871	Taiwan	3.4	2.8	1.2–6.4	0.015	[51]
		Platelet, < 150 × 10^3^/mm^3^	1000	Europe and Canada	5.7	1.1	1.0–1.1	0.029	[61]
		Albumin, < 35 g/dL	399	Sweden	7.8	4.4	1.3–14.7	0.016	[69]
		Liver cirrhosis, yes	1351	Taiwan	4.0	8.4	4.1–17.0	<0.001	[70]
		Liver cirrhosis, yes	4663	Canada	5.6	3.2	1.2–9.0	–	[71]
	Post-SVR								
		FIB-4 index, high	522	Japan	7.2	1.7	1.1–2.9	0.02	[55]
		APRI ≥ 0.7	1351	Taiwan	4.0	2.9	1.5–5.7	0.002	[70]
		Elastography liver stiffness > 12 kPa	376	Taiwan	7.6	6.3	2.1–19.5	0.001	[56]
		Liver cirrhosis, yes	10,738	USA	2.8	6.7	4.3–10.4	<0.001	[36]
		Platelet, < 130 × 10^3^/mm^3^	571	Japan	9.0	3.9	1.5–10.1	0.004	[72]
	Age, years	≥50	562	Japan	4.8	4.1	1.4–17.4	<0.01	[53]
		≥55	571	Japan	9.0	3.6	1.4–9.6	0.009	[72]
		≥60	642	Taiwan	4.4	3.7	1.3–10.2	0.012	[52]
		≥60	871	Taiwan	3.4	3.8	1.7–8.4	0.001	[51]
		≥60	4663	Canada	5.6	4.4	1.3–15.3	–	[71]
		>60	1094	Japan	4.2	3.1	1.3–6.6	0.009	[58]
		>60	1056	Japan	4.7	3.1	1.3–7.4	0.01	[68]
		>60	1000	Europe and Canada	5.7	9.8	1.2–77.8	0.031	[61]
		≥65	1425	Japan	3.3	5.8	1.1–30.1	0.036	[54]
		≥65	1351	Taiwan	4.0	2.7	1.2–6.3	0.017	[70]
		≥65	10,738	USA	2.8	4.5	2.0–10.4	<0.001	[36]
		Older	589	Taiwan	4.7	1.1	1.0–1.1	0.046	[73]
	Sex	Male	1094	Japan	4.2	12.0	2.8–50.0	<0.001	[58]
		Male	4663	Canada	5.6	3.3	1.1–9.6	–	[71]
		Male	571	Japan	9.0	7.6	1.7–33.1	0.007	[72]
	Diabetes	Yes	522	Japan	7.2	2.1	1.1–4.0	0.045	[55]
		Yes	376	Taiwan	7.6	4.0	1.3–12.1	0.021	[56]
		Yes	399	Sweden	7.8	3.2	1.1–9.6	0.035	[69]
		Yes	10,738	USA	2.8	1.9	1.2–2.9	0.005	[36]
		Yes	1000	Europe and Canada	5.7	2.3	1.0–5.3	0.057	[61]
		Yes	4663	Canada	5.6	1.6	0.6–4.0	–	[71]
		Yes	589	Taiwan	4.7	3.8	1.4–10.1	0.008	[73]
	Elixhauser comorbidity index	Yes (≥1)	4663	Canada	5.6	2.2	1.0–5.1	–	[71]
	Alpha-fetoprotein, ng/mL								
	Pre-SVR	≥8	562	Japan	4.8	2.6	1.2–6.1	<0.05	[53]
		≥15	1351	Taiwan	4.0	1.9	1.0–3.6	0.038	[70]
		≥20	871	Taiwan	3.4	3.2	1.6–6.2	0.001	[51]
	Post-SVR	≥5	1425	Japan	3.3	8.1	2.7–23.9	<0.001	[54]
		≥5	571	Japan	9.0	3.6	1.4–9.6	0.009	[72]
		≥15	1351	Taiwan	4.0	2.3	1.0–5.3	0.043	[70]
		≥10	1094	Japan	4.2	7.8	2.9–16.8	<0.001	[58]
	Race/ethnicity	Hispanic	10,738	USA	2.8	2.3	1.1–4.8	0.032	[36]
	Alcohol abuse	Yes	562	Japan	4.8	3.9	1.7–9.0	<0.01	[53]
		Yes	10,738	USA	2.8	1.7	1.1–2.6	0.021	[36]
		Yes	4663	Canada	5.6	1.1	0.34–3.3	–	[71]
	Illicit drug use	Yes	4663	Canada	5.6	3.7	1.0–14.3	–	[71]
	AST	>100 IU/L	1056	Japan	4.7	3.1	1.3–7.3	0.01	[68]
	AST/ALT ratio	>0.72	1000	Europe and Canada	5.7	1.0	1.0–1.1	0.068	[61]
	GGT	>75 U/L	642	Taiwan	4.4	6.4	2.2–18.9	0.001	[52]
Viral factor	Genotype 1b with Gln70 (His70) variant		1273	Japan	1.1	10.5	2.9–38.2	<0.001	[34]
	Genotype 3		10,738	USA	2.8	1.6	1.0–2.7	0.071	[36]
	Genotype 3		4663	Canada	5.6	1.4	0.58–3.4	–	[71]

All data are from interferon-based studies

*APRI* aspartate aminotransferase-to-platelet ratio index, *ALT* alanine aminotransferase, *AST* aspartate aminotransferase, *FIB-4* fibrosis-4, *GGT* gamma-glutamyl transpeptidase, *HCV* hepatitis C virus, *HCC* hepatocellular carcinoma, *SVR* sustained virologic response

Current practice guidelines recommend regular biannual HCC screening for cirrhotic patients with active HCV infection, but it is still undetermined whether and how post-SVR patients should be monitored for future HCC development and if any of the risk-associated variables has clinical utility [1]. Molecular hallmarks of persisting HCC risk in post-SVR livers may serve as biomarkers to identify a subset of patients still at risk and should be therefore monitored by regular HCC screening. A pan-etiology HCC risk-predictive gene signature in the liver, which was shown to predict post-SVR HCC development, may serve as a biomarker to identify a subset of SVR patients who should be regularly monitored for future HCC [38].

### Post-DAA HCC development and recurrence

Accumulating clinical experience of DAA-based treatment has suggested that post-SVR HCC development and recurrence may be more frequent compared to interferon-based treatment (Table 2). In a small series of HCC patients who achieved an SVR by all-oral DAAs after HCC treatment, tumor recurrence rates of approximately 30% within 6 months were reported; these rates are alarmingly high, however, the observation period was short, a proper control group was lacking, and the finding was not replicated in a subsequent study [39–43]. Further studies are needed to clarify whether DAAs increase HCC incidence and to determine the natural history and baseline post-SVR HCC incidence according to the type of anti-HCV therapy in each specific patient population. Interestingly, chronic hepatitis B patients treated with directly-acting anti-HBV drugs, entecavir or other nucleos(t)ide analogues, showed higher HCC incidence compared to peg-interferon-treated patients, suggesting that the difference in HCC-suppressive effect may be a common phenomenon across different hepatitis viruses [44].

Several studies suggested a possibly distinct difference in host immune modulation between interferon and DAAs. Rapid decline of HCV viral load by DAAs was experimentally or clinically associated with restored HCV-specific, often exhausted, CD8+ T cell function, memory T cell re-differentiation and lymphocyte deactivation, and normalized NK cell function [45–48], all of which may indicate a quick loss of anti-HCV immune responses. Interestingly, reactivation of other co-infected viruses, such as herpes virus, was observed after DAA-based anti-HCV therapy [49], suggesting simultaneous loss of bystander immune response to the viruses and possibly to neoplastic cells, which may lead to higher HCC recurrence after DAA treatment. On the other hand, complete remission of follicular lymphoma after DAA-based therapy was reported, suggesting that the influence of DAA-based SVR on cancer may vary according to cancer types and biological/clinical contexts [50].

## Conclusions

HCV-related HCC will remain a major health problem in the coming decades despite the clinical deployment of DAAs. Access to the new generation antiviral therapies should be substantially improved to achieve meaningful prognostic benefit at the population level. The development of a vaccine remains an important goal for global control and eradication of infection. Post-SVR HCC is an emerging problem, with urgent unmet needs for the clinical strategy of early tumor detection and intervention, as well as elucidation of its molecular mechanisms for therapeutic target and biomarker discovery. Prolonged clinical observation should be further accumulated to determine the impact of DAA-induced SVR on HCC development and recurrence as well as on other cancer types.
